# Antioxidant and Cytotoxic Activities and Phytochemical Analysis of *Euphorbia wallichii* Root Extract and its Fractions

**Published:** 2012

**Authors:** Ihsan Ul-Haq, Nazif Ullah, Gulnaz Bibi, Simab Kanwal, Muhammad Sheeraz Ahmad, Bushra Mirza

**Affiliations:** a *Department of Biochemistry, Faculty of Biological Sciences, Quaid-i-Azam University (45320), Islamabad, Pakistan.*; b *Department of Biochemistry, PMAS-Arid Agriculture University Rawalpindi, Rawalpindi Pakistan.*

**Keywords:** Antioxidant, Cytotoxicity, DNA protection, *E. wallichii*, Phytochemical analysis

## Abstract

*Euphorbia wallichii *a perennial herb growing mainly in Himalayas has been widely used in folk medicines for its medicinal properties. In the present study, the crude methanolic root extract (CME) and its fractions; n-Hexane Fraction (NHF), n-Butanol Fraction (NBF), Chloroform Fraction (CHF), Ethyl acetate Fraction (EAF) and Aqueous Fraction (AQF) of this plant specie were investigated for antioxidant and cytotoxic activities and phytochemical analysis. Antioxidant activity was determined by using 2,2-diphenyl-1-picryl-hydrazyl free radical (DPPH) and DNA protection assay performed on pBR322 plasmid DNA. In both these assays, promising results were obtained for CME as well as other fractions. The IC_50_ values for DPPH assay were in a range of 7.89 to 63.35 μg/ml in which EAF showed the best anti-oxidant potential and almost all the tested samples showed certain level of DNA protection. The cytotoxic activity was assessed by using Sulforhodamine B (SRB) assay on human cell lines; H157 (Lung Carcinoma) and HT144 (Malignant Melanoma). The IC_50_ values of the tested samples ranged from 0.18 to 1.4 mg/mL against H157 cell line whereas against HT144 cell line the IC_50_ values ranged from 0.46 to 17.88 mg/mL with NBF fraction showing maximum potential for both. Furthermore, the phytochemical analysis of CME and its fractions showed the presences of flavonoids, saponins, tannins, terpenoides and cardiac glycosides with varying concentrations.

## Introduction

The Euphorbiaceae Family is one of the largest families of higher plants, about 300 genera and 7,500 species have been reported (1, 2). There are about 2,160 species in genus *Euphorbia *and it is regarded as the 6^th^ largest genus among flowering plants. Many plants of this genus have been used in folk medicine for centuries against various diseases including infections, skin diseases as well as cancer (1-5). 

*E. wallichii *is a perennial herb growing mainly in the Himalayas, including Qinghai-Tibetan Plateau area of China, India, Nepal, and Kashmir. The roots of this plant have been conventionally used in folk medicine for treatment of edema, skin disease, cutaneous anthrax and exanthema (6, 7). A chemical literature survey of *E. wallichii*, showed the presence of abietane (8), diterpenoids (6, 7) and triterpenoid of the multiflorane class (1). Recently Ali *et al*. (2) reported significant results for *in vitro *phytotoxicity, cytotoxicity and antibacterial activity of *E. wallichii *root extract and fractions. Plants of the genus *Euphorbia *are known to possess considerable anti-cancer (7), anti-tumor and antioxidant (7, 9) potential. However antioxidant and cytotoxic activity (towards cancer cells) of *E. wallichii *in comparison to its phytochemicals have not been reported previously. This provoked us to investigate *E*. *wallichii *for its cytotoxic and antioxidant activity along with phytochemical studies.

## Experimental

*Plant material*

The roots of *E. wallichii *were collected from Mushkpuri tract, Nathia Gali, N.W.F.P. Pakistan in July 2008. The plant was identified by taxonomist Dr Rizwana Aleem Qureshi, Associate Professor, Department of Plant Sciences, Quaid-i-Azam University, Islamabad, Pakistan with reference to “Flora of Pakistan” and comparing with already identified herbarium sheets preserved in the herbarium. A voucher specimen (Specimen No. 125755) was deposited in ISL Herbarium, Quaid-i-Azam University, Islamabad Pakistan.

*Extraction and fractionation*

Fresh roots of the *E. wallichii *were washed, sliced and dried under shade and ground. The root extract was prepared in analytical grade methanol (5 kg in 12 L) for 72 h, then methanol was removed and residue was immersed in methanol for further five days. Thereafter, the methanol was decanted and filtered with filter paper. The filtrate was subsequently concentrated under reduced pressure at 45°C in rotary evaporator (Rotavapor R-200 Buchi, Switzerland) and dried to a constant weight (700 gram) in vacuum oven at 45°C (Vacucell, Einrichtungen GmbH). This was called crude methanolic root extract (CME).

The CME was then subjected to fractionation, where 230 g of CME was suspended in 200 mL of distilled water. This aqueous suspension was further subjected to solvent-solvent extraction for five fractions, namely; *n*-Hexane Fraction (NHF), *n*-Butanol Fraction (NBF), Chloroform Fraction (CHF), Ethyl acetate Fraction (EAF) and aqueous Fraction (AQF). The overall fractionation procedure is given in flowchart diagram (Figure 4). 

*Biological activities*

*Determination of antioxidant activity*

The free radical scavenging activity was measured by using 2,2-diphenyl-1-picryl-hydrazyl free radical (DPPH) assay. DPPH assay was performed according to the procedure described by Kulisic *et al. *(10) modified by Obeid *et al. *(11). DPPH solution was prepared by dissolving 3.2 mg DPPH in 100 mL of 82% methanol. 2800 μL of DPPH solution was added to glass vials followed by the addition of 200 μL of CME solution in Methanol; leading to the final concentration of 100 μg/mL, 50 μg/mL, 25 μg/mL, 10 μg/mL, 5 μg/mL, 2 μg/mL and 1 μg/mL. Mixtures were shaken well and kept in dark at controlled room temperature (25°C-28°C) for one hour. Absorbance was measured at 517 nm by using spectrophotometer (DAD 8453, Agilent). Methanol (82%) was used as blank while mixture of 200 μL of methanol and 2800 μL of DPPH solutions were taken as negative control. Ascorbic acid was used as positive control. Each test was performed in triplicates and percentage inhibition was measured according to formula given below and IC_50_ values were calculated by graphical method.

Scavenging effect (%) = [(Ac-As)/Ac] x100 

Where “Ac” means Absorbance of negative control and “As” means Absorbance of test sample. In order to determine the antioxidant activity of different fractions the same procedure was then repeated with all of the fractions *i.e*. NBF, NHF, EAF and AQF.

*DNA protection assay*

To study the effects of CME and its fractions on plasmid DNA the procedure of Tian and Hua (12), modified by Nawaz *et al. *(13) was adopted. The reaction was conducted in an Eppendorf tube at a total volume of 15 μL containing following components; 0.5 μg pBR322 DNA suspended in 3 μL of 50mM phosphate buffer (pH 7.4), 3 μL of 2 mM FeSO_4_, 5 μL of tested samples (CME and its fractions) and 4 μL of 30% H_2_O_2_. Resulting mixture was incubated at 37°C for 1 h and was subjected to 1% agarose gel electrophoresis for 1 h at 100 volts. DNA bands (supercoiled, linear, and open circular) were stained with ethidium bromide and were qualitatively analyzed by scanning with Doc-IT computer program (VWR). Evaluations of antioxidant or prooxidant effects on DNA were based on the increase or loss percentage of supercoiled monomer, compared with the control value. To avoid the effects of photoexcitation of samples, experiments were done in the dark and untreated supercoiled DNA, supercoiled DNA treated with 2 mM FeSO_4_, supercoiled DNA treated with 30% H_2_O_2_ and supercoiled DNA treated with 2 mM FeSO_4_ + 30% H_2_O_2_ were used as control along with the test samples. 

*Cytotoxic activity by sulforhodamine B (SRB) assay*

The human cancer cell lines H157 (lung carcinoma) and HT144 (malignant melanoma) were cultured in RPMI1640 media (Gibco BRL, Life Technologies, Inc) supplemented with 10% heat inactivated fetal bovine serum in a humidified incubator at 37°C with 5% CO_2_. The cells were subcultured approximately once every four days by 98% trypsin EDTA solution (pH 7.2). Growth inhibition of H157 and HT144 cells was determined by using the modified SRB assay as described by Skehan *et al. *(14). Briefly, cells were seeded at a density of 5×10^3^ cells/well in 96-well plates. After 24 h, serial dilutions of samples (CME and fractions) and standard drug (Methotrexate) solutions were added for each concentration. The cells were exposed to test samples and drugs for continuous 72 h. For cell fixation, the culture medium was removed and trichloroacetic acid (50%, 100 μL) was added in each plate. Then the plates were air-dried and 0.4% SRB (sigma) in 1% acetic acid was added for 30 min and unbound dye was washed out with 1% acetic acid. After air-drying, SRB dye within cells were dissolved with 100 μL solution of tris-base 10mM (pH 10.5). The optical density of the extracted SRB dye was measured with a microplate reader (Platos R 496) at 490nm. The 50% inhibitory concentration (IC_50_) of the test drugs was calculated using a Probit analysis program. Chemosensitivity of H157 and HT144 cells transfected with control vector was determined by SRB assay as described above. 

*Phytochemical analysis*

The crude methanol extract and its fractions were screened phytochemically for the presence of tannins, alkaloids, saponins, flavonoids, steriods phlobatannins, terpenoids and cardiac glycosides by standard methods of phytochemical analysis (15-19). For total flavonoid determination ammonium chloride chlorimetric method was used (20). The total phenolic contents were determined according to Velioglu *et al*. (21) method and Folin-Ciocalteu reagent was used.

## Results and Discussion

*Biological activities*

*Antioxidant activity*

DPPH free radical scavenging assay was used to evaluate antioxidant potential of our samples. The DPPH scavenging assay is a reliable and easy method for antioxidant property evaluation. This assay has been used for investigating antioxidant properties of wheat, vegetables, herbs, edible seed oils, conjugated linoleic acids, and flours in several different organic solvent systems including ethanol, aqueous acetone, methanol, aqueous alcohol, and benzene (9, 22-25).

CME as well as fractions showed effective free radical scavenging activity as determined by DPPH assay. The results of free radical scavenging assay are given in Table 1. 

**Table 1:** Percentage scavenging and IC_50_ of antioxidant assay for CME and Fractions of *E. wallichii *root

**S. No.**	**Extract**	Scavenging effect (%) at different concentrations ± STDEV *							**IC**_50_ **μg/mL**
		**100 μg/mL**	**50 μg/mL**	**25 μg/mL**	**10 μg/mL**	**5 μg/mL**	**2 μg/mL**	**1 μg/mL**	
**1.**	**CME**	92.6 ± 0.23	93.0 ± 0.23	93.0 ± 0.32	53.0 ± 0.21	44.0 ± 0.00	18.0 ± 0.32	6.0 ± 0.56	8.410
**2.**	**NHF**	12.2 ± 0.13	-	-	-	-	-	-	>100
**3.**	**NBF**	93.1 ± 0.60	93.2 ± 0.72	93.0 ± 0.52	37.0 ± 0.23	34.2 ± 0.34	3.1 ± 0.02	-	13.55
**4.**	**CHF**	90.2 ± 0.72	31.1 ± 0.42	9.1 ± 0.35	-	-	-	-	63.35
**5.**	**EAF**	92.7 ± 0.56	93.7 ± 0.45	91.3 ± 0.35	54.1 ± 0.34	48.0 ± 0.25	29.1 ± 0.21	5.0 ± 0.13	7.89
**6.**	**AQF**	91.0 ± 0.32	90.2 ± 0.26	90.1 ± 0.76	29.0 ± 0.59	5.8 ± 0.32	-	-	15.23
**7. **	**A.A.**	95.0 ± 0.53	94.8 ± 0.61	90.0 ± 0.48	66.4 ± 1.2	44.7 ± 0.23	30.0 ± 0.41	11.7 ± 0.71	5.63

*Data represents mean values of three replicates, A.A.: Ascorbic acid, - : shows no activity

Crude extract showed promising antioxidant activity with IC_50_ value of 8.41 μg/mL while EAF has maximum antioxidant activity with IC_50_ value of 7.89 μg/mL. Other fractions; NBF, AQF and CHF have IC_50_ values of 13.55, 15.23 and 63.35 μg/mL respectively, while NHF showed lowest free radical scavenging activity and has IC_50_ >100 μg/mL. EAF has excellent free radical scavenging with IC_50_ 7.89 μg/mL which is comparable to ascorbic acid, a very important antioxidant compound (26). Phytochemical assay of the EAF shows that it has high concentrations of tannins which are known to be potent antioxidants (27). The presence of tannins in EAF may be responsible for its antioxidant activity. Our phytochemical analyses (Table 3) also support this hypothesis as antioxidant activity varies with the change in tannins concentration.

Transition metal iron, in human, may react with H_2_O_2_ to produce ^●^OH trough a Fenton like reaction. The ^●^OH is the most influential oxidizing agent among other reactive oxygen radicals, and it can oxidize biomolecules` including DNA, protein, lipid, and carbohydrate (27,28,29). Oxidative DNA damage from reactive oxygen species (ROS) can play a serious role in many biological processes including mutagenesis, aging and carcinogenesis (31-33).

**Figure 1:**
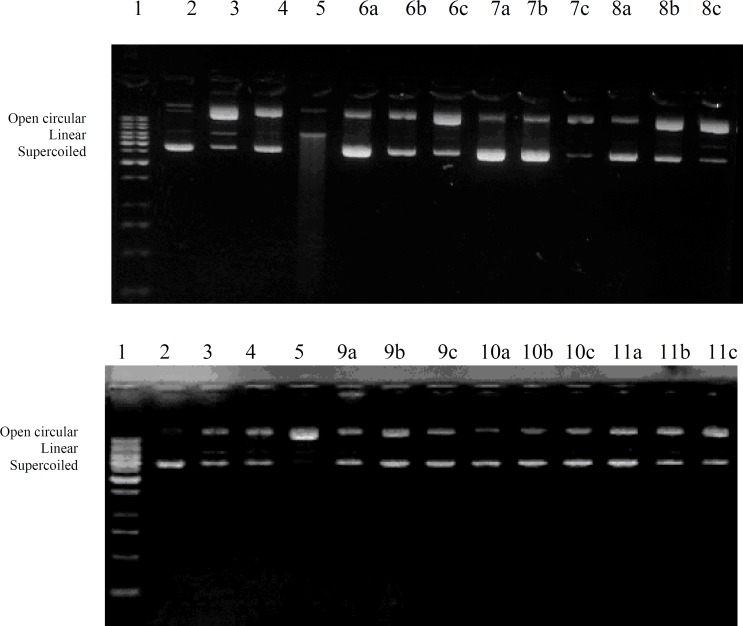
DNA protection affect of crude extract and different fractions of *E. wallichii *roots.1 = 1Kb DNA Ladder, 2 = Plasmid DNA (pBR 322), 3 = Plasmid DNA treated with FeSO_4_, 4 = Plasmid DNA treated with H_2_O_2_, 5 = Plasmid DNA treated with FeSO_4_ and H_2_O_2_, a= 1000 μg/mL, b = 100 μg/mL, c = 10 μg/mL, 6 = CME, 7 = NHF, 8 = NBF, 9 = CHF, 10 = EAF, 11 = AQF in which a,b,c shows three replicates for each test sample

Results of DNA protection assay demonstrated that CME has concentration dependent DNA protection properties (Figure 1). Among other fractions NHF has good DNA protection activity 

at 1000 μL/mL and 100 μL/mL while weak protection activity at 10 μL/mL. NBF also has good protection activity at 1000 μL/mL while moderate to weak protection activity at 100 μL/mL and 10 μL/mL respectively. CHF and EAF have good protection activity at all tested concentrations, while AQF has concentration dependent DNA protection activity as shown in the Figure 1. 

*Cytotoxic activity*

Infectious diseases and cancer have a primary position in use of medicinal plants for drug discovery and 60-75% anticancer and antiinfectious drugs have origin from natural products (34). The connotation in discovery of natural drugs for cancer chemoprevention is rapidly increasing (35-37). Several plant species rich in phenolics and flavonoids are reported for anticancer therapeutic properties. For cytotoxic activity, the SRB assay was conducted on CME of *E. wallichii *and its fractions by using human cell lines H157 (Lung Carcinoma) and HT144 (Malignant Melanoma). NBF shows the highest cytotoxicity on H157 cell line and has IC_50_ value 0.18 mg/mL followed by EAF at 0.22 mg/mL. The IC_50_ value of CME was 0.29 mg/mL against this cell line whereas standard drug Methotrexate has shown IC_50_ value 5.9E-5 mg/mL. Against Malignant Melanoma (HT144) cell line similar results were obtained. In this test the cytotoxic effect of NBF was highest with IC_50_ value of 0.46 mg/mL followed by EAF fraction having IC_50_ of 0.54 mg/mL and CME having IC_50_ value of 0.87 mg/mL. Standard drug Methotrexate has shown IC_50_ value 0.02 mg/mL for this cell line as shown in Table 2.

**Table 2:** Cytotoxic activity of CME and Fractions of *E. wallichii *root *.

**Extract Nam**e	**Treatment (mg/mL) against H157 cell line**					**IC**_50 _ **mg/mL**	**Treatment (mg/mL) against HT144 cell line**					**IC**_50_ **mg/mL**
	**0.1**	**0.5**	**1**	**2.5**	**5**		**0.1**	**0.5**	**1**	**2.5**	**5**	
CME	38 ± 0.9	56 ± 1.0	64 ± 0.6	73 ± 0.7	75 ± 0.8	0.29	20 ± 0.8	45 ± 1.1	58 ± 1.1	63 ± 1.2	67 ± 1.1	0.87
NHF	38 ± 0.7	51 ± 1.0	54 ± 1.1	54 ± 0.6	58 ± 0.9	0.74	11 ± 0.9	15 ± 0.8	30 ± 0.9	38 ± 0.9	38 ± 0.9	17.88
NBF	41 ± 0.9	61 ± 2.0	73 ± 0.7	75 ± 0.9	76 ± 0.9	0.18	28 ± 1.0	54 ± 0.9	66 ± 1.1	68 ± 1.3	74 ± 0.8	0.46
CHF	29 ± 0.7	33 ± 2.1	49 ± 1.1	55 ± 1.1	65 ± 1.1	1.36	7 ± 1.1	28 ± 1.1	48 ± 1.3	60 ± 1.1	61 ± 1.1	1.69
EAF	42 ± 1.1	57 ± 1.0	67 ± 1.2	76 ± 1.1	78 ± 1.1	0.22	37 ± 1.1	43 ± 1.1	57 ± 1.2	64 ± 1.1	75 ± 1.1	0.54
AQF	22 ± 0.7	34 ± 0.8	44 ± 1.1	57 ± 0.7	66 ± 0.9	1.4	7 ± 0.9	17 ± 0.9	27 ± 0.9	33 ± 0.9	45 ± 0.9	11.7
Methotrexate	81 ± 0.9	92 ± 1.1	97 ± 1.1	98 ± 0.3	98 ± 0.7	5.9E-5	61 ± 0.9	67 ± 1.1	69 ± 0.9	74 ± 1.3	85 ± 1.2	0.02

*a) Activity given as % inhibition of cancer cells. b) H157 (lung carcinoma cells) and HT144 (malignant melanoma cells). c) IC_50_ was calculated by graphical method using MS-Excel 2007.

Cytotoxic activity recorded in the present study seems in accordance with our finding of phytochemicals in this plant species (Table 3). Since the phytochemical evaluation indicated the presence of flavonoids in CME and its fractions particularly in EAF, NBF and AQF which also shows promising cytotoxic potential. Among these active fractions NBF and EAF have highest cytotoxic and antioxidant potential. As mentioned earlier, these fractions have higher phenolic contents as well (Table 3).

**Table 3:** Phytochemical analysis of CME and Fractions of *E. wallichii*

**S/No **	**Costituents/ test**	**Extracts / Fractions**					
		**CME**	**NHF**	**NBF**	**CHF**	**EAF**	**AQF**
1	Alkaloids						
	Dragendoff's	+	-	-	-	-	-
	Mayer's	+	-	-	-	-	-
	Wagners	+	-	-	-	-	-
2	Flavonoids						
	Harborne	++	+	++	+	++	+
3	Steriods						
	Liebermann-Burchard reaction	-	-	-	-	-	-
4	Saponins						
	Frothing Test	+	-	++	+	++	+++
5	Tannins						
	FeCl_3_ Test	+++	-	+++	-	+++	+++
6	Phlobatannins	-	-	-	-	-	-
7	Terpenoids	+++	+++	-	++	-	-
8	Cardiac Glycosides						
	Keller-Kiliani Test	+++	+++	-	++	-	-

+ : weekly present, ++: moderately present, +++: highly present , - : not present

The presence of tannins at high or moderate level in NBF, EAF and AQF and in CME may also be responsible for their promising antioxidant/DNA protection and cytotoxic activity. Khan *et al *(38) reported that, “tannins exhibit a stronger anti-oxidant effect by their ability to quench hydroxyl radical and singlet oxygen mediated DNA cleavage”. Therefore, the present work on antioxidant/DNA protection or cytotoxic activity and phytochemical analysis of *E. wallichii *can be a good start for isolation of valuable secondary metabolites from root extract of this plant.

*Phytochemical analysis*

Phytochemical analysis showed the presence of different classes and concentrations of compounds in CME and fractions (Table 3). Analysis shows the presence of flavonoids, saponins, tannins, terpenoids and cardiac glycosides whereas alkaloids were completely absent. On fractionation these compounds distributed in solvents of different polarities on the basis of solubility as shown in Table 3. Moderate to high concentrations of saponins, tannins and cardiac glycosides were present in CME and its fractions.

*The flavonoid contents*

**Figure 2:**
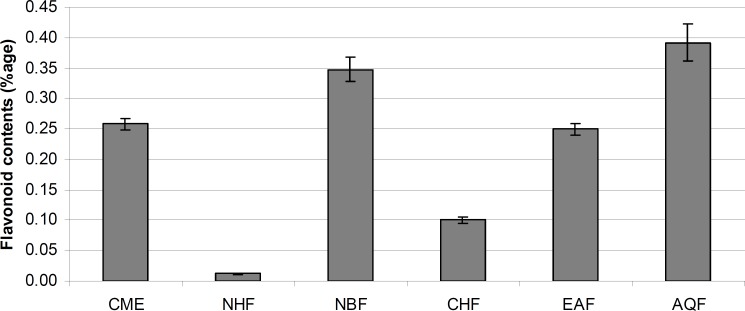
Flavonoid contents (% w/w dry extract/fraction) of *E. wallichii *CME and its fractions with standard deviations

The total flavonoid contents of the CME and its fractions were determined in terms of flavonoids percentage as shown in Figure 2. In CME the flavonoid contents were 0.26%. Among fractions, the highest flavonoid content were found in AQF (0.39%) followed by NBF (0.35%), EAF (0.25%), CHF (0.10%) and NHF (0.01 %). It is well documented that the flavonoids show antioxidant activity and have considerable effects on human health and nutrition (39, 40). 

*The phenolic contents*

**Figure 3:**
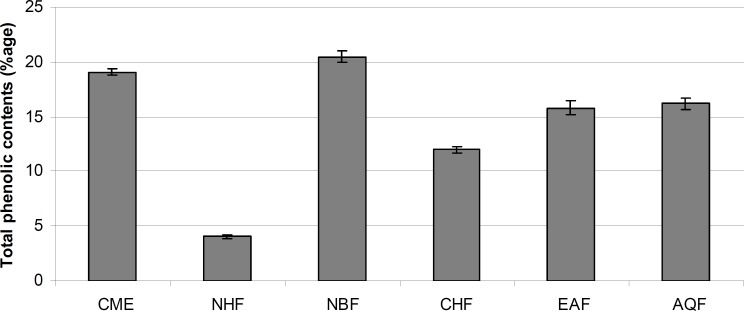
Total phenolic contents (% w/w of dry extract/fraction) of *E. wallichii *CME and its fractions with standard deviations

The total phenolic contents of the CME and its fractions are shown in Figure 3. Among CME and its fractions, NBF fraction has highest phenolic content (20.49%), followed by CME fraction (19.08%), AQF (16.18%), EAF (15.81%), CHF (12.01%) and NHF (4.03%). The relationships between phenolic content of medicinal plants and antioxidant activity is well documented (21, 41). 

**Figure 4:**
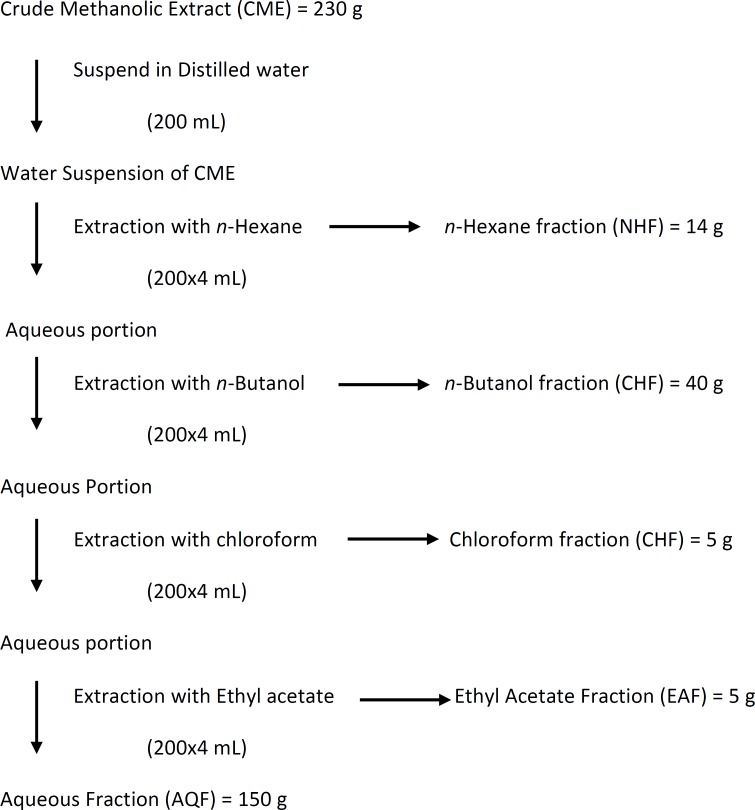
Schematic diagram for fractionation of crude methanolic root extract (CME).
